# Field evaluation of a blood based test for active tuberculosis in endemic settings

**DOI:** 10.1371/journal.pone.0173359

**Published:** 2017-04-05

**Authors:** Aasia Khaliq, Resmi Ravindran, Syed Fahadulla Hussainy, Viwanathan V. Krishnan, Atiqa Ambreen, Noshin Wasim Yusuf, Shagufta Irum, Abdul Rashid, Muhammad Jamil, Fareed Zaffar, Muhammad Nawaz Chaudhry, Puneet K. Gupta, Muhammad Waheed Akhtar, Imran H. Khan

**Affiliations:** 1 College of Earth and Environmental Sciences, University of the Punjab, Lahore, Pakistan; 2 Department of Pathology and Laboratory Medicine, University of California, Davis, California, United States of America; 3 Department of Computer Science, School of Science and Engineering, Lahore University of Management Sciences, Lahore, Pakistan; 4 Department of Chemistry, California State University, Fresno, California, United States of America; 5 Gulab Devi Chest Hospital, Lahore, Pakistan; 6 Department of Pathology, Allama Iqbal Medical College, Lahore, Pakistan; 7 NextGen In Vitro Diagnostics, New Delhi, India; 8 School of Biological Sciences, University of the Punjab, Lahore, Pakistan; Fundació Institut d’Investigació en Ciències de la Salut Germans Trias i Pujol, Universitat Autònoma de Barcelona, SPAIN

## Abstract

Over 9 million new active tuberculosis (TB) cases emerge each year from an enormous pool of 2 billion individuals latently infected with *Mycobacterium tuberculosis* (*M*. *tb*.) worldwide. About 3 million new TB cases per year are unaccounted for, and 1.5 million die. TB, however, is generally curable if diagnosed correctly and in a timely manner. The current diagnostic methods for TB, including state-of-the-art molecular tests, have failed in delivering the capacity needed in endemic countries to curtail this ongoing pandemic. Efficient, cost effective and scalable diagnostic approaches are critically needed. We report a multiplex TB serology panel using microbead suspension array containing a combination of 11 *M*.*tb*. antigens that demonstrated overall sensitivity of 91% in serum/plasma samples from TB patients confirmed by culture. Group wise sensitivities for sputum smear positive and negative patients were 95%, and 88%, respectively. Specificity of the test was 96% in untreated COPD patients and 91% in general healthy population. The sensitivity of this test is superior to that of the frontline sputum smear test with a comparable specificity (30–70%, and 93–99%, respectively). The multiplex serology test can be performed with scalability from 1 to 360 patients per day, and is amenable to automation for higher (1000s per day) throughput, thus enabling a scalable clinical work flow model for TB endemic countries. Taken together, the above results suggest that well defined antibody profiles in blood, analyzed by an appropriate technology platform, offer a valuable approach to TB diagnostics in endemic countries.

## Introduction

*Mycobacterium tuberculosis* (*M*. *tb*.), the etiologic agent of tuberculosis (TB), is one of the most successful human pathogens on the planet. Approximately two billion people are infected with *M*. *tb*. worldwide. Of these, between five to fifteen percent develop active TB in their lifetime, and each year about 1.5 million die[1]. In approximately 10% of TB cases, non-pulmonary disease may occur, involving various organ systems with or without lung involvement[2,3]. TB is generally curable with drug therapy provided it is diagnosed correctly and in a timely manner[4,5]. Because TB symptoms are strikingly similar to other pulmonary diseases common in TB-endemic countries (e.g., chronic obstructive pulmonary disease, COPD), patients suspected of TB may need to be tested in numbers five to ten times larger than actual TB patients, thus, further taxing the limited capacity of health care systems for accurate diagnosis of TB[4]. It is estimated that of 9 million new TB cases worldwide in 2013, about 3 million were not even accounted for[6].

The infected host typically mounts a vigorous immune response to *M*. *tb*. that in most individuals may lead to a latent infection[7,8]. The tuberculin skin test (TST) and the interferon-γ release assay (IGRA) can detect such immune responses in individuals with latent TB[9,10]. For active TB in high burden countries diagnostics are primarily focused on sputum smear acid-fast bacilli (AFB) microscopy and chest X-ray (CXR). AFB-microscopy is subjective, inconsistent, and sensitivity can be as low as 30% in many TB endemic countries (globally 30–70%), resulting in a large number of undetected cases [6]. In addition, sputum smear microscopy is at times unable to distinguish between tuberculous and non-tuberculous mycobacteria and therefore, specificity may range from as low as 93% to as high as 99%[11–15]. Radiographic appearance of TB is not uniform and is left to interpretation by the clinician and/or radiologist such that sensitivity ranges from 67–77%, with specificity about 50%[13,16]. The bacterial culture method (solid and liquid) is the gold standard for detection of *M*. *tb*.in patient sputum. The major drawback of the cumbersome solid culture procedures is that *M*. *tb*. is a slow growing organism, requiring about 4–8 weeks to obtain results. Culture has been estimated to confirm 80–85% of TB cases, but with a high specificity of 98%[17]. Several molecular-based diagnostic methods for detecting *M*. *tb*. in sputum samples have been introduced over the last several years but with marginal success in capacity building in endemic countries to deal with high patient burden[18–22].

Among high TB burden countries, Pakistan ranks 5^th^, and accounts for 61% of this burden in the WHO Eastern Mediterranean Region [23]. The major obstacle in treatment and eradication of the disease is the delayed diagnosis due to unawareness, poor clinical facilities, and lack of health care infrastructure[24]. In such resource limited settings poor detection rates lead to significant underdiagnosis and mismanagement of infectious cases, and possible development of drug resistance. In endemic countries like Pakistan and India, the lack of affordable, rapid and precise diagnostic tools has led to the use of a large number (73) of commercial serological tests; 60 tests in the lateral flow (immunochemistry) format and 13 in the ELISA format[25]. Serology tests for active TB are based on antibody recognition of antigens of *M*. *tb*. The premise is that the presence of specific *M*. *tb*. antibodies in the blood is an indirect indication of active TB disease. Key to accurate diagnosis using serology is the correct choice of antigen(s) that are able to differentiate between active disease and latent infection while maintaining high sensitivity and specificity. The WHO issued a negative recommendation on existing serology tests based on a systematic evidence-based process[26–31]. It is important to note that the WHO was careful to mention that the negative recommendation only applied to serology tests that were currently on the market, stating that “the WHO policy encourages research to develop new serological tests for TB based on antigen/antibody biomarkers and the negative recommendation only applies to existing commercial tests”[27, 32–37].

We reasoned that challenges related to conventional serology tests can be overcome by the multiplex diagnostic method that employs multiple *M*. *tb*. antigens for efficient and reliable serodiagnosis. We reported the development of multiplex microbead immunoassay (MMIA) for serodiagnosis of *M*. *tb*. in non-human primates[38], and further preclinical studies demonstrated proof-of-concept of this approach[39]. For human applications, clinical samples were first analyzed to determine the best *M*. *tb*. antigens for use in the MMIA[40]. Here we demonstrate the utility of a multiplex serodiagnostic panel of 11 *M*. *tb*. antigens. The application of this multiplex system on plasma or serum samples is cost effective with an estimated commercial price of under US$8 for the end-user. This active TB detection system enables an efficient and scalable clinical model with a capacity to handle 1 to 360 patients per day in endemic settings.

## Materials and methods

### Clinical workflow at the patient recruitment site

Patients were recruited at Gulab Devi Chest Hospital (GDCH), Lahore, Pakistan from 2012–2015. GDCH follows WHO general guidelines for TB diagnostics, directly observed treatment short course (DOTS), and patient care. Patients positive by acid-fast bacilli sputum smear microscopy (AFB microscopy) for at least one sample are considered positive (AFB^+^), and their anti-TB treatment (ATT) is immediately initiated. For AFB^-^ cases, the patient is prescribed broad spectrum antibiotics (Amoxicillin 500mg and Co-trimoxazole combined with Trimethoprim, 100mg) for 2 weeks, followed by another round of AFB microscopy and CXR. If the CXR is suggestive and the clinical symptoms consistent with pulmonary TB persist, the patient is considered a AFB^-^ pulmonary TB patient, and ATT is initiated. In this study, for all pulmonary TB cases, sputum samples were cultured (Lowenstein-Jensen (LJ) & MGIT) for confirmation of TB.

### Diagnostic tests

Sputum samples were processed for AFB-microscopy (Ziehl-Neelsen (ZN) staining) at two places: Microbiology Laboratories at Jinnah Hospital, Allama Iqbal Medical College (AIMC), and GDCH Lahore. Cultures were performed on two independent sputum samples from the patients who were prescribed ATT at these two diagnostic laboratories. At AIMC, sputum cultures were performed by two methods: 1) on solid LJ media and 2) liquid MGIT-960. At GDCH, only solid culture on LJ media was performed. A rapid HIV testing kit (Advance Quality Rapid Anti-HIV (1 & 2) Test Card (whole blood/serum/plasma) by Intec Products Inc. Xiamen, China; Catalog Number: ITP02002) was used for HIV testing in TB and COPD patients. All pulmonary disease patients included in this study were HIV negative by this method. It is important to note that Pakistan is among the lowest HIV prevalence (general population) countries worldwide, and the negative HIV results in this study are consistent with this information[41].

### Sample groups

Blood and sputum samples from patients, and healthy individuals (blood samples only) were obtained under the protocols approved through the Institutional Review Boards (IRB) at the School of Biological Sciences (SBS), University of the Punjab, Lahore. Per country’s immunization policies, all individuals in this study were vaccinated with BCG[42].

#### (1) Pulmonary disease groups

Blood and sputum samples were collected from AFB ^+^ TB patients (n = 100), and AFB^-^ TB patients (n = 124). A written informed consent was obtained from each patient. Data including gender, age, clinical history and physical characteristics were recorded by a trained technician. The patients (median age 26 years; IQR: 20, 40) were diagnosed with active pulmonary TB on the basis of positive result for sputum smear AFB-microscopy, CXR suggestive of TB, and physicians’ assessment based on clinical presentation including persistent cough for more than two weeks, and other systemic symptoms when present, *e*.*g*., fever (low grade and intermittent), weight loss, night sweats *etc*. Confirmation of TB was made on the basis of LJ and MGIT culture results.

One category of patients included the AFB^+^ and culture positive patients (Culture^+^) (n = 100); the second category included the AFB^-^ /Culture^+^ patients (n = 101); and the third category included AFB^-^ culture negative (Culture^-^) patients (declared as active TB patients based on both clinical symptoms and CXR results) (n = 23). TB for AFB^-^/Culture^-^ group was further confirmed by clinical follow up in a complete physical examination (weight gain, any signs of cough or fever, improvement in appetite) and CXR, at the conclusion of successful completion of 6 months of ATT. At the end of treatment, sputum examination was done even if only saliva was available (cured patients may or may not produce sputum) to assess negative results. All patients were thoroughly interviewed and examined by a panel of physicians. All patients in this group, receiving ATT, showed a positive response to treatment, and their clinical and radiological findings substantially improved at the end of ATT as compared to the time of diagnosis, confirming that they were TB patients.

Chronic obstructive pulmonary disease (COPD) patients with median age of 60 years (IQR: 52, 63) of mixed sex (n = 55), were included as a disease control group. In TB endemic countries, like Pakistan and India, cigarette smoking is common and is the leading cause of COPD.

A majority of non-TB, pulmonary disease patients (about 60 to 70%) seen for suspicion of TB at GDCH and AIMC, suffer from either COPD or bronchial asthma; roughly half falling into each category. TB physicians involved in this study (authors MJ, AR, NWY and SI), each with over a decade of clinical experience, find that COPD is clinically more challenging to differentiate from TB, and the key symptoms can be very similar (e.g., excessive sputum production, hemoptysis, fatigue, and unintended weight loss), and thus, may lead to misdiagnosis. Other pulmonary diseases are less common (e.g., lung cancer, bronchiectasis, Brucellosis etc.), or seasonal and acute (e.g., pneumonia–typically December to February). COPD patients were available in numbers suitable for the computational analysis in this study. COPD patients in this study tested negative for TB (AFB and culture).

#### (2) Healthy control group

Blood samples of the healthy individuals (n = 79) of mixed sex (median age 21 years; IQR: 20, 22) were taken from the same geographical area as the TB patients; these individuals had no history of active TB, no pulmonary symptoms and no known medical conditions (infection, cancer, or metabolic disease). This group consisted of random, young individuals to represent the general healthy population for comparison to TB patients. Because Pakistan is among the lowest HIV prevalence countries worldwide, these overtly healthy individuals were not tested for HIV status[41]. The antibody responses in these individuals were intact since cross-reactive antibodies against several antigens, especially Rv3507, Rv1009, Rv1099 (see S1 Appendix), were prominent. The detection of such antibodies in healthy individuals was the reason these antigens were rejected. Furthermore, we evaluated total plasma IgG levels for which the MFI values typically ranged between 6000–7000 consistently across all individuals suggesting similar IgG production (S1 Appendix).

### Blinded pilot study in India

A pilot study was performed at the National Institute for Research in Tuberculosis (NIRT), at Chennai, under the guidelines of Indian Council for Medical research (ICMR), New Delhi. Archived serum samples obtained for study were approved by institutional ethical committee established by the National institute for Research in Tuberculosis (NIRT), Chennai. All subjects provided written informed consent before drawing the blood. All the study subjects were adults. All subjects were confirmed HIV negative by routine AIDS tests. These archived serum samples from 78 healthy individuals (median age 30 years; IQR: 23, 37) and 74 confirmed TB patients (median age 37 years; IQR: 27, 48) were randomized, de-identified and coded, and provided for analysis (gift from Dr. Alamelu Raja, Department of Immunology, NIRT). Such samples are routinely provided for pilot studies to assess the pre-commercialization performance of novel blood-based TB diagnostic tests that are presented to ICMR for consideration. All 74 samples from TB patients were sputum culture positive (AFB^+^ = 63; AFB^-^ = 11).

### Blood sample collection, processing, and storage

As previously described, blood samples (5 ml) were collected into a Vacutainer tube (EDTA, catalog # 367899; BD, Franklin Lakes, NJ) *via* venipuncture, and plasma was collected and frozen in aliquots at −80°C until use[40]. Patients were de-identified (no personal information), and the stored plasma samples were shipped on dry ice for analysis at the University of California, Davis.

### Sputum sample processing

All sputum samples were processed for liquefaction and decontamination by a conventional mycobacterial N-acetyl L-cysteine sodium hydroxide (NaOH-NALC) method[43–45]. After this decontamination procedure, aliquots were collected for *M*. *tb*. AFB detection by ZN staining, inoculation of MGIT tubes used for the BACTEC^TM^ MGIT^TM^ 960 system, solid culture on LJ media and for extraction of total DNA[46].

### DNA isolation and *M*.*tb*. confirmation through IS6110 PCR

DNA isolation from culture samples was done by the CTAB method[47]. The polymerase chain reaction (PCR) was performed to amplify the IS6110 sequence of 200 base pairs using DNA extracted from culture as previously described[48].

### Multiplex plasma-antibody analysis

As previously described, recombinant antigens from 28 *M*. *tb*. genes were expressed in *Escherichia coli* and purified[38]. A multiplex microbead immunoassay (MMIA) based on the xMAP technology platform (Luminex Corp, Austin, TX) was designed to detect the plasma antibodies using uniquely labeled microbeads conjugated with the following recombinant *M*.*tb*. antigens (Rv3881c, Rv0934 (P38 or PstS1), Rv2031c (HspX), Rv1886c (Ag85b), Rv1860 (MPT32), Rv3874 (CFP10), Rv3875 (ESAT6), Rv3804c (antigen 85a [Ag85a]), Rv3418c (GroES), Rv3507, Rv1926c, Rv3874-Rv3875 (CFP10-ESAT) fusion, Rv2878c, Rv1099, Rv3619, Rv1677, Rv2220, Rv2032, Rv1984c (CFP21), Rv3873, Rv0054, Rv3841 (Bfrb1), Rv1566c, Rv2875 (MPT70), Rv0129c (Ag85c), Rv1009, Rv1980c (MPT64), and Rv0831c. These antigens are designated in this paper as A1, A2, A3, A4, A5, A6, A7, A8, A12, A13, A14, A15, A16, A17, A18, A19, A20, A21, A22, A23, A24, A25, A26, A27, A28, A29, A30, A31, respectively. In addition, uniquely labeled microbeads were coated with membrane extracts (MEM) from H37RV, HN878, CDC1551 *M*. *tb*. strains (designated as A9, A10, and A11) obtained from the TB Resource Center at Colorado State University (Fort Collins, CO) [49]. The assay was performed as previously detailed[40].

### Antibody data

Data were collected as median fluorescence intensity (MFI), as previously described[40]. The total number of samples used in this study was 356 including TB and COPD patients, and healthy individuals. Data for 31 antibodies were collected from each sample (in duplicate), resulting in a total of 22,072 data points. All data underlying the findings in this study are presented in the S1 Appendix that contains antibody data for all groups in separate labeled sheets in Excel file.

### Data visualization

Data were visualized using box and whisker plots by the package ‘ggplot2’ in RStudio version 3.2.2. In addition, cluster analysis of data was performed to visualize the antibody profiles in all samples using R-Studio version 3.2.2, limma (linear models for microarray data) package, and g plots (graphic plots) package. Firstly, Quantile normalization procedure was used to scale the log_2_ ratios for all patients TB relative to the COPD patients and healthy group for MFI levels of each antigen in each sample[50]. Secondly, all samples were clustered using hierarchical clustering with ‘ward.2’ distance method and represented in the heat map by dendrograms.

### Data analytics: Overview

Multivariate analysis was performed on multiplex data to obtain the fold changes (and p-values) of each antibody in TB patients as previously described[39, 40, 51, 52]. High fold changes indicated value of an antigen for discrimination between TB and non-TB cases. To classify samples into TB and non-TB we used the following 6 classification algorithms: Decision Tree, k Nearest Neighbor, Logistic Regression, Naïve Bayes, Random Forest and Support Vector Machines. Standard accuracy metrics highlighted Decision Tree and Random Forest as the top two performing algorithms. Lastly, since the conventional algorithms do not provide individual cutoff for each antigen, the Decision Tree algorithm was optimized following the principles described by Ohta et al. [53].

#### A. Multivariate analysis of antibody data to determine fold changes

Fold changes (by Multivariate analysis) enabled the identification of antibodies for which patterns were significantly different in patients compared to the control groups as previously detailed [49, 40, 51, 52]. Fold changes in TB patients compared to control groups were calculated across different categories of TB patients (AFB^+^/Culture^+^, AFB^-^/Culture^+^, and AFB^-^/Culture^-^) [52].

#### B. Classification algorithms

The following classification algorithms, which are typically used in computational biology, were employed: Decision Tree, k Nearest Neighbor, Logistic Regression, Naïve Bayes, Random Forest and Support Vector Machines. Antibody data for all antigens were analyzed with three-fold cross-validation for classification purposes.

Three-fold cross-validation approach randomly divides the original data into randomly selected datasets with approximately equal number of samples [54]. The classification algorithms to analyze data were used such that in one instance, two of the three datasets were used as the training sets and the third one as the test set (*e*.*g*., datasets A & B, A & C, and B & C). The models from these training sets were tested on the corresponding test sets by each of the six classification algorithms.

#### C. Optimized Decision Tree algorithm and cut off determinations (see Results why Decision Tree was selected for optimization)

In the classification done by the conventional Decision Tree algorithm, the tree is grown by binary splitting of a node (an antibody) into branches (children) above or below a cutoff point that gives the maximum information gain *i*.*e*. minimizes the impurity (false positives) of the children nodes[55]. This approach does not completely account for the biological behavior of antibodies in TB patients since not all patients contain antibodies against particular antigens, and some patients may have antibodies against only a single antigen. The conventional algorithm continues to compute all nodes, adding an unnecessary noise to the analysis and thus results in a suboptimal performance. Therefore, the conventional Decision Tree algorithm was optimized such that for each antibody, the cutoff which discriminated between TB patient samples and controls with the highest precision (*i*.*e*., no false positives) was used[53]. This approach enabled the determination of whether a sample was positive or not for TB, even if a single antibody was detected. The cutoffs of the antigens that yielded the highest precision and greatest number of true positives were recorded at all binary splitting of nodes by the optimized algorithm. The following steps summarize how the process at each node of the tree occurs:

Step 1: For each antigen in the training set, the MFI values that discriminate the samples with highest TB patient precision (the lowest number of false positives) would be determined.
Step 2: The MFI values from Step 1 which would have the greatest number of true positives would be branched further (split into left (non-TB) and right (TB) children). That particular MFI value would be selected as the cutoff for a particular antigen.
Step 3: If the right child (> = cutoff value) from Step 2 is a pure set (100% precision–contains no false positives) then it need not be split further, while the left child would be used as input for Step 1 of the next iteration. If the right child is not a pure set (i.e., contains false positives), the computational process for that particular iteration would end.

Once the full-grown tree was obtained on every training set, the cutoffs recorded for all the antigens were used and the model was tested on the test-sets, under the three-fold cross validation approach[54]. Another algorithm that performed as well as Decision Tree, or better, was Random Forest. It is impractical to attempt to optimize Random Forest for the applications relevant to this report because the analysis involves a combination of multiple decision trees, with a restrictive default feature set in every iteration [50].

### Performance of classification algorithms

The prediction power of each model was evaluated by test efficiency (TE; also referred to as test accuracy) and Mathew Correlation Coefficient (MCC) [56]. TE and MCC are mathematically defined as follows:

$$TE=\frac{TP+TN}{TP+TN+FP+FN}$$

$$MCC=\frac{TP\times TN-FP\times FN}{\sqrt{\left(TP+FP)\times (TP+FN)\times (TN+FN)\times (TN+FP\right)}}$$

Data classification yielded the following measures for the multiplex serology test: true positive (TP), true negative (TN), false positive (FP), and false negative (FN), and accuracy Metrics: $\mathrm{Positive}\;\mathrm{Predictive}\;\mathrm{Value}\;(\mathrm{PPV})=\frac{TP}{TP+FP}$, $\mathrm{Negative}\;\mathrm{Predictive}\;\mathrm{Value}\;(\mathrm{PPV})=\frac{TN}{TN+FN}$.

### Antibody data analysis in India

Multiplex antibody assay was performed as in Pakistan but with the following exceptions: i) seven *M*.*tb*. antigens instead of 31 were available for the multiplex analysis in India (A1, A2, A3, A4, A9, A5, and A6), ii) archived serum samples instead of plasma were available, and iii) due to the limited multiplex antigen panel (7 antigens), computer modeling analysis that was optimized using Pakistani data with 31antigens could not be applied. Cut-off values were calculated for the 7 antigens from data in healthy individuals for Indian and Pakistani samples separately (Cutoff = Mean MFI + (3 × standard deviation). The cut-off values were used to determine antibody positive samples (at least one antibody) in the data sets from the two TB endemic countries for comparison.

## Results

### Characterization of TB patients and control subjects

A majority of TB patients in this study were positive by culture, PCR for IS6110 (DNA isolated from culture), and had CXR findings suggestive of TB. Of 224 TB patients, 199 (88.8%) were positive by LJ or MGIT, or both, at least at one of the two hospitals (S1 Table). A total of 100 patients (44.6%) were positive by AFB microscopy (AFB^+^). Of all AFB^+^ patients (n = 100), 98 were also positive by culture (2 AFB^+^ patients were found to be culture negative at both hospitals—excluded from the analysis). Thus, 98 of 224 (43.75%) of TB patients were categorized as AFB^+^/Culture^+^. A total of 101 (45%) patients were negative by AFB microscopy at both sites but were positive by culture (AFB^-^/Culture^+^). Several AFB^-^ patients confirmed to have TB (see below) were in addition culture negative (LJ and MGIT), and thus represented a group of patients negative by microbiological analysis of sputum (n = 23) but were determined to be TB patients and were prescribed ATT (see Materials and Methods for details).

### Data visualization: TB patients and controls

Raw data (log_2_ MFI), were plotted as box and whisker plots for overall comparison of values of each antigen among the patient categories (AFB^+^/Cul^+^, AFB^-^/Cul^+^, and AFB^-^ /Cul^-^) and controls (healthy individuals and COPD patients) (S1 Fig, S2 Fig and S3 Fig). MFI values in patients for many antigens were higher in TB patients compared to both sets of controls. Overall, discriminatory antibodies between TB patients and controls (*e*.*g*., A1, A2, A3, A4, A5, A6, A7, A8 etc.) had differences between TB patient categories in the descending order from AFB^+^/Cul^+^ category to AFB^-^/Cul^+^, and to AFB^-^ /Cul^-^. However, for each category, the differences between TB patients and each of the control groups (Healthy and COPD) were very similar.

**Fig 1:**
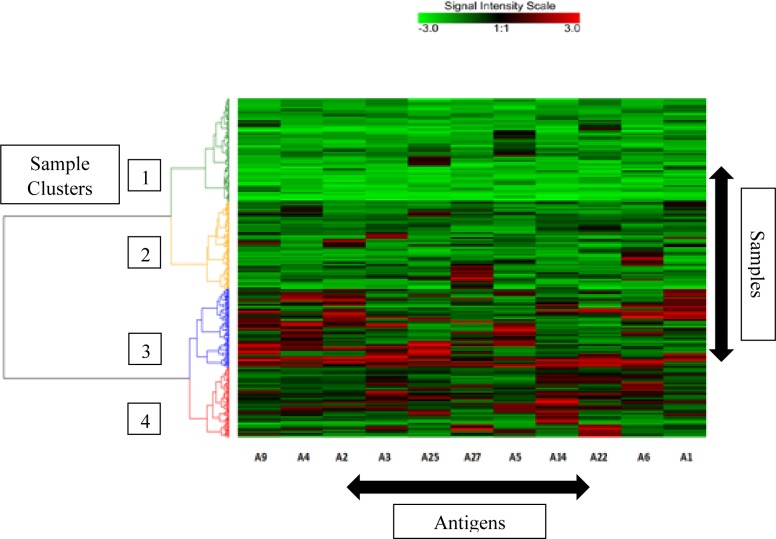
Natural groupings (clusters) of study population based on antibodies. against eleven selected *M*.*tb*. antigens (see Table 1). The signal intensity in arbitrary units ranges from “-3 to 3” after scaling log_2_ ratios of the MFI values for antibodies with green as the minimum and red as the maximum signal intensities. The extent of the signal for each antibody in each sample is depicted by this intensity scale. Sample clusters are indicated by color-coded dendrogram on the left side of the heat map; the four clusters are numbered for clarity.

Data were also visualized by cluster analysis of natural grouping of samples (AFB^+^ and AFB^-^ TB patients confirmed by culture, and Healthy and COPD controls) for an overall depiction of antibodies against 11 antigens selected on the basis of computational classification (see below). Samples were clustered, as depicted in a heat map that displays antibodies and sample groups (Fig 1). Samples represented by Cluster 1 predominantly contained healthy individuals and COPD patients, while Cluster 3 and Cluster 4 comprised predominantly of TB patients. However, Cluster 2, which largely contains controls (healthy and COPD), also depicts mixed sub-clusters with a few TB patients, both of which contained samples with low antibody responses, as we have previously reported [48].

### Multivariate analysis of antibody data to determine fold changes

Levels of antibodies in different categories of patient groups were calculated as fold changes of MFI between TB patients and healthy and COPD patient controls through multivariate analysis of antibodies. The three individual categories of TB patients (AFB^+^/Cul^+^, AFB^-^/Cul^+^, and AFB^-^ /Cul^-^) separately, and the combined AFB^+^/Cul^+^, and AFB^-^/Cul^+^ groups contained significantly higher levels of antibodies compared to healthy and COPD controls (S2 Table). The elevation in antibody responses in the three individual categories of TB patients above and control groups followed the descending trend with the greatest fold increases in AFB^+^/Cul^+^ category followed by AFB^-^/Cul^+^, and AFB^-^ /Cul^-^, as noted above.

Performance of algorithms for classification of TB patients: Six algorithms (Decision Tree, k Nearest Neighbor, Logistic Regression, Naïve Bayes, Random Forest and Support Vector Machines) were compared for classification of samples based on detection of antibodies to *M*.*tb*. antigens. For TB patient categories confirmed by culture, the results revealed that Decision Tree and Random Forest had best overall performance followed by Logistic Regression, Support Vector Machines, k Nearest Neighbor and Naïve Bayes (S3 Table).

**Fig 2:**
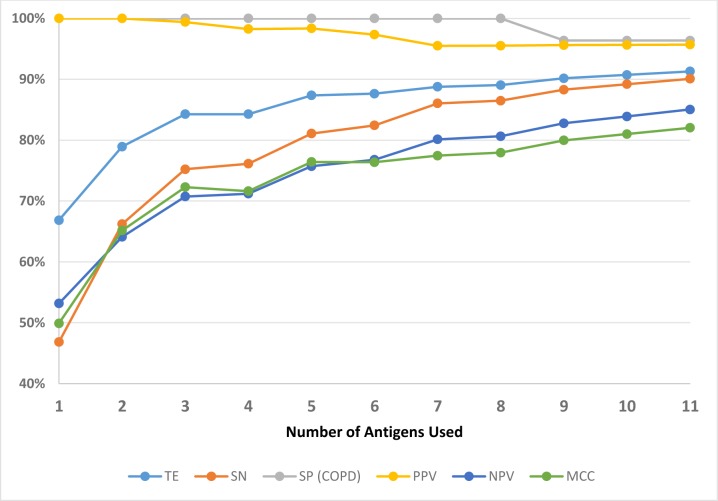
Accuracy matrix for the number of antigens used in the multiplex panel from 1 to 11 (selected *M*.*tb*. antigens), as analyzed by the Optimized Decision Tree algorithm. SN = Sensitivity, PPV = Positive Predictive value, NPV = Negative Predictive Value, SP = Specificity, TE = Test Efficiency, and MCC = Mathew Correlation Coefficient.

Performance of the optimized Decision Tree algorithm: The optimized Decision Tree algorithm afforded a superior performance and highlighted a more precise set of 11 antigens (out of 31 antigens) which facilitated the best classification performance in comparison to the other algorithms as noted by improved metrics: TE, MCC, sensitivity, specificity, PPV, and NPV (Fig 2). The optimized algorithm also enabled identification of a range of antigens, and showed improvement in yield of test metrics with sequential increase in the number of these *M*. *tb*. antigens from 1 to 11 (Table 1). Importantly, the improved performance revealed that the number of antigens can be reduced to 11 from a total of 31 for accurate detection and classification of TB to yield a high-performance diagnostic test for all patients, and for the three individual groups of TB patients (Table 1). This analysis also showed that by most criteria, the sensitivity of multiplex serology test would be compromised by reducing the number of antigens below 11 in the multiplex serodiagnostic panel (Fig 2).

**Table 1:** Impact of the number of antigens on performance metrics determined by modified decision Tree algorithm. Results in two combined categories of TB patients (AFB^+^/Culture^+^ & AFB^-^/Culture^+^) for sensitivity, positive and negative predictive values, test efficiency (TE) and Matthew Correlation Coefficient (MCC), and test sensitivity in individual categories of TB patients (AFB^+^/Culture^+^, AFB^-^/Culture^+^, AFB^-^/Culture^-^), and test specificity (COPD patients and healthy group). Values shown are percentages.

Antigens	Antigens used	AFB^+^/Culture^+^ & AFB^-^/Culture^+^ n = 199					SN (AFB+C+) n = 98	SN (AFB-C+) n = 101	SN (AFB-C-) n = 23	SP (COPD) n = 55	SP (HBP) n = 79
		SN	PPV	NPV	TE	MCC					
A1, A2, A3, A4, A5, A6, A9, A14, A22, A25, A27	11	91	96	86	92	83	95	88	87	96	91
A1, A3, A4, A5, A6, A9, A12, A14, A22, A27	10	90	97	85	92	83	94	87	87	96	94
A1, A3, A4, A5, A6, A9, A14, A22, A27	9	88	97	83	91	82	93	84	87	96	95
A1, A3, A4, A5, A6, A9, A22, A27	8	85	99	80	90	81	92	79	78	100	99
A1, A4, A5, A6, A9, A22, A27	7	84	99	79	90	81	92	78	78	100	99
A1, A4, A5, A9, A22, A27	6	83	99	78	89	80	91	77	78	100	99
A1, A4, A5, A9, A22	5	82	99	77	88	79	91	75	74	100	99
A1, A4, A5, A9	4	80	99	75	87	77	89	74	70	100	99
A1, A5, A9	3	77	99	72	85	74	88	69	65	100	99
A1, A9	2	69	99	66	80	67	83	59	52	100	99
A1	1	47	100	53	67	50	54	42	39	100	100

Performance of the TB multiplex serology test: Samples from healthy individuals were used to calculate the cut-off values for anti-*M*.*tb*. antibody detection for the multiplex microbead serodiagnostic test for TB by the optimized Decision Tree algorithm. Plasma samples from COPD were used to determine the assay specificity. By including 11 *M*. *tb*. antigens as determined above, sensitivity for combined categories of TB patients (smear positive and negative, all culture positive) and specificity (healthy, and COPD samples) of the multiplex serology test, were 91% and 96% respectively (Table 1). In individual categories of TB patients, test sensitivities followed a descending order (with antigen number increasing from 1 to 11) from AFB^+^/Cul^+^, to AFB^-^/Cul^+^, and to AFB^-^ /Cul^-^ (Table 1). Sensitivity, TE, and MCC displayed progressive improvement with the increase in number of selected antigens from 1 to 11 (Table 1). These criteria did not improve beyond the eleven selected antigens. A small decrease in specificity in COPD patients beyond 8 antigens was observed but remained at 96% (Table 1).

### Blinded pilot study in India: Comparison of sensitivity and specificity of the TB multiplex diagnostic system in two endemic countries

The samples provided by NIRT were serum, a suboptimal alternative since the multiplex immunoassay test had been optimized for plasma. Another difference was that a multiplex panel of only 7 antigens was available in India for this pilot project. Samples in which at least 1 antibody out of 7 was detected were considered positive. The multiplex assay results obtained in India were directly compared to the results in samples from Pakistan (for the same 7 antigens), to compare test performance for samples from both countries (Table 2). Overall, the results for combined AFB^+^ and AFB^-^ TB samples were similar for sensitivity (86%, and 84%), specificity (93.7%, and 90%), PPV (95.5%, and 89%) and NPV (80.1%, and 85%) for Pakistan and India, respectively. However, for AFB^+^ samples, sensitivity in plasma samples from Pakistan was about 9% higher (92.9%) than the serum samples from India (84%). This difference is probably because the samples available in India were archived samples stored frozen (-80^°^C) for several years with variable duration for different samples, while the Pakistani samples were used within a year of storage (-80^°^C).

**Table 2:** Blinded study in India: sensitivity and specificity compared to Pakistan, using 7 antigens.

	^$^Pakistan					^$^India				
Patient Category	N	SN (%)	^*^SP (%)	PPV	NPV	N	SN (%)	^*^SP (%)	PPV	NPV
**Combined TB Patients (AFB**^**+**^**Cul**^**+**^ **& AFB**^**-**^**Cul**^***+***^**)**	199	86	93.7	95.5	80.1	74	84	90	89	85
**AFB**^**+**^**Cul**^**+**^	98	92.9				63	84			
**AFB**^**-**^**Cul**^**+**^	101	82.2				11	81.8			

^$^Plasma samples were used in Pakistan, and serum samples in India (plasma not available). ^*^Specificity was determined in Pakistan (n = 79) and India (n = 78) using indigenous healthy individuals in each country.

## Discussion

In TB endemic countries, the frontline TB diagnostic test, AFB microscopy, suffers from low and variable sensitivity. Culture is sparingly used because of the long wait time for results. As a consequence, the percentage of bacteriologically confirmed cases in most TB endemic countries is underwhelming. For example, in 2014, of all TB patients, only 52% in Pakistan and 66% in India were bacteriologically confirmed; the rest, 48% and 34%, respectively, depended solely on clinical diagnosis [1]. Clinical diagnosis in turn depends on the training and experience of the physician and quality of CXR findings suggestive of TB, both of which vary widely from country to country, and clinic to clinic. Here we report that among AFB negative patients, who would have not have been bacteriologically confirmed under standard clinical practice, the sensitivity multiplex serology test was 88% (n = 101). The test detected 95% (n = 98) of the AFB positive patients. In combined AFB positive and negative patients, all confirmed by culture, the sensitivity was 91%. In addition, our multiplex serology test detected 87% of active TB patients that were negative by sputum based bacteriological methods (AFB microscopy, MGIT and LJ culture; n = 23).

The multiplex serology test described in this report is user-friendly, accurate, flexible and scalable to analyze samples ranging from 1 to 360 samples (one machine) from pulmonary TB suspects per day. In TB endemic countries like India and Pakistan, chest diseases hospitals and clinics have high patient volumes that typically require handling 150–200 TB suspects per day. This multiplex serodiagnostic test can handle high patient volume, and is cost effective for developing countries with an estimated price of under $8 that compares favorably to the cost of AFB microscopy ($6–9, for 2–3 sequential tests). With overall sensitivity of 91%, and specificity of 91% (healthy population) and 96% (COPD patients), the multiplex serology test exceeds the performance of AFB microscopy that may miss about half the patients. However, COPD patients tend to be older (than TB patients, as in this study). We examined the antibody responses in 24 TB patients that were older than 55 years of age (12 in AFB^+^/Cul^+^ group with median age 65; and 12 in AFB^-^/Cul^+^ and AFB^-^/Cul^-^ groups with median age 65). Antibody responses detected in them resulted in TB detection sensitivities of 92% and 83%, respectively, similar to all TB patients a majority of whom were young. These results suggest that antibody production in older individuals was as intact as in the young. Furthermore, we evaluated total plasma IgG levels for which the MFI values typically ranged between 6000–7000 consistently across all individuals, in all groups, suggesting that antibody responses in older individuals were similar to the younger ones (S1 Appendix).

The turn-around time for the multiplex serodiagnostic is less than a day, which is a substantial improvement over the minimum of two days needed for AFB microscopy. It is important to note that on the ground in most TB endemic countries, the reality is that AFB microscopy’s turn-around time is several days to weeks. During this time, the patients await treatment, and may continue to spread the disease. A test with higher throughput, such as this multiplex serology test, can drastically improve the work flow at TB hospitals/clinics in endemic countries by providing reliable and relatively rapid results for hundreds of TB suspects in a single day as opposed to several days. Our model for TB detection can significantly decrease the work load at diagnostic laboratories, while reducing the reliance on both subjectivity and technical labor.

The optimal number of *M*.*tb*. antigens for the performance of the multiplex serology test was determined to be 11. These results demonstrate that TB serology tests must include several *M*. *tb*. antigens instead of one or two. This is consistent with previous work by us and others demonstrating that several, but a manageable number, of *M*. *tb*. antigens in a multiplex format is needed for high performance of serology tests [37, 40]. The increase in the number of antigens, as well as their combination in a multiplex test, must be optimized systematically, without a significant sacrifice in specificity (Table 1). In this study, we investigated the value of antibody profiling in the AFB^+^/Cul^+^ patients, as in our previous proof-of-concept studies [40]. Importantly, we have extended our investigations to include two additional critical groups of TB patients, AFB^-^/Cul^+^ and AFB^-^ /Cul^-^, where sputum based frontline TB diagnostic test (AFB-microscopy) failed to produce a result, as is the case in about half the TB patients (AFB-microscopy has approximately 50% sensitivity). Furthermore, we have optimized computational analysis that was previously focused only on highlighting the antigens for which antibodies had the highest fold-increase in signal, in TB patients [40]. Eight antigens (Rv3881, Rv0934, Rv0054, Rv3804c, Rv2031c, Rv1886c, Rv0129c, Rv1860 and Rv1980) were highlighted by the computational analysis to be the top tier (Tier-1) in the previous study [40]. In the present study, computational analysis was performed by optimized Decision Tree algorithm to mark a sample positive for TB if antibodies against even a single antigen were detected regardless of how high a signal (as long as significant at p-Value < 0.01) was produced. Accordingly, eleven antigens were highlighted (p-Value <0.01) which maximized the test sensitivity without compromising specificity (Rv3881, Rv0934, Rv2031c, Rv1886c, Rv1860, Rv3874, Rv2875, Rv3841, Rv1926c, MEMH37Rv, and Rv1984). Of these 11, 5 antigens were the same as previously highlighted in Tier-1 and were the most prevalent among TB patients with highest fold-increase in signal (Rv3881, Rv0934, Rv2031c, Rv1886c, and Rv1860). For another 4 antigens the overall signal was not necessarily as high as that for Tier-1antigens but because the overlap with antibodies against the first 5 antigens, and between each other was minimum, they improved the test sensitivity (Rv3874, Rv2875, Rv3841, and Rv1926c). These 4 antigens were previously considered of low priority and categorized among the 8 antigens in Tier-2[40]. Two additional antigens, Rv1984 and MEMH37Rv, were also highlighted as significant (p-Value <0.01) and valuable since antibodies against them did not overlap substantially with other antibodies, thus contributing to the increase in test sensitivity. It is also important to note that although many of the antigens used in our test have been reported previously for TB diagnostic applications (e.g. by ELISA, protein chip array, lateral flow etc.), the multiplex microbead array configuration used in this report is different and may have resulted in differences in sensitivity and specificity of these (individual or in combinations) antigens[34, 57–59]. This multiplex test is based on coupling of unique fluorescent microbeads (5–6 μm) such that the *M*. *tb*. antigens are exposed on the entire surface of the bead making them more accessible for antibody binding as opposed to a planar configuration of other platforms such as ELISA, protein chip, membrane in lateral flow system etc. This feature may, therefore, represent a configurational advantage of our multiplex serology test. The robustness of our multiplex serology test was demonstrated in a blinded study performed in India using archived serum samples from TB patients and controls. The multiplex test performance in India on serum samples was similar to the results of studies performed in Pakistan on plasma samples despite the fact that the test was optimized, and antibody baselines were established, using plasma samples. Although the number of antigens available for the blinded pilot study in India was lower (7 antigens) than the optimal number determined in Pakistani studies (11 antigens), the results highlighted two important features: 1) performance of the multiplex test in serum samples is overall comparable to plasma samples, and 2) the test performance is robust even under completely different sampling conditions in a different TB endemic country. Furthermore, we have obtained similar consistent results in a clinical study performed on samples collected in Uganda (manuscript in preparation, these authors). Taken together, the results of the blinded study in India, and studies in Pakistan, demonstrate the utility, reproducibility in different endemic country settings, as well as flexibility in sample composition (serum or plasma). In addition, we have performed studies with dried blood spots (DBS) collected on filter paper, in parallel with plasma samples in Pakistan. Studies to validate the DBS method are ongoing in both Pakistan and India. Future studies are also focused on a large scale clinical trial of the multiplex serology test (11 selected antigens) under the guidance of ICMR in India. Plans to study plasma and DBS samples from 2500 pulmonary TB suspects (approximately 500 individuals are expected to be confirmed for pulmonary TB) have been submitted to the Drug Controller General of India (DCGI), New Delhi.

The multiplex serology test has a demonstrated ability to achieve consistent and high levels of sensitivity and specificity. Sensitivity exceeds that of AFB microscopy, and compares favorably to culture and published data on GenXpert (S4 Table). Importantly, we show that high variability in sensitivity and specificity of the existing commercial TB serology tests that lead to a negative recommendation by WHO, can be overcome by the multiplex serology approach. This is accomplished by: 1) the use of several antigens instead of one or two, and 2) a robust multiplex microbead suspension array technology. The multiplex serodiagnostic test is rapid (approximately 2 hours) with increased throughput (360 samples per 8-hour day with a single instrument), accurate, and low cost (under US $8 to the patient). The cost includes sample preparation, and instrument (Luminex MagPix) lease cost; available in many developing countries (purchase or lease through suppliers e.g., EMD Millipore, or directly from Luminex - www.luminexcorp.com). Furthermore, the multiplex serology test is flexible and scalable to handle anywhere from 1 to 360 samples per day, with a potential for even higher throughput with the addition of robotics. This throughput capacity is in contrast to the low throughput of AFB microscopy, culture and GenXpert.

## Supporting information

S1 Table Summary of the sputum AFB microscopy and culture results in TB patients. (DOCX)

S2 Table Fold changes of antibodies in TB patients compared to controls by multivariate analysis. (DOCX)

S3 Table Performance of six classification algorithms compared to optimized Decision Tree. (DOCX)

S4 Table Comparison of sensitivity and specificity of the Multiplex TB Serodiagnostic Panel containing 11 antigens to established sputum-based tests. (DOCX)

S1 Appendix All raw data. (XLSX)

S1 Fig Anti-*M*. *tb*. antibody levels in AFB^+^/Culture^+^ TB patients compared to controls. Box and whiskers representation of log_2_ MFI values of antibodies against different *M*.*tb*. antigens. In each box plot, the central line inside the box represents the median. The box depicts the interval between 25% and 75% percentiles. Whiskers indicate the range of data spread while small circles show outliers. A. AFB^+^/Culture^+^ TB patient data (n = 98) are represented by red boxes, and blue boxes represent data for healthy individuals (n = 79), for each antigen. B. AFB^+^/Culture^+^ TB patient data (n = 98) are represented by red boxes, and green boxes represent data for COPD patients (n = 55), for each antigen.
(PDF)

S2 Fig Anti-*M*. *tb*. antibody levels in AFB^-^/Culture^+^ TB patients compared to controls. Box and whiskers representation of log_2_ MFI values of antibodies against different *M*.*tb*. antigens. In each box plot, the central line inside the box represents the median. The box depicts the interval between 25% and 75% percentiles. Whiskers indicate the range of data spread while small circles show outliers. A. AFB^-^/Culture^+^ TB patient data (n = 101) are represented by red boxes, and blue boxes represent data for healthy individuals (n = 79), for each antigen. B. AFB^-^/Culture^+^ TB patient data (n = 101) are represented by red boxes, and green boxes represent data for COPD patients (n = 55), for each antigen.
(PDF)

S3 Fig Anti-*M*. *tb*. antibody levels in AFB^-^/Culture^-^ TB patients compared to controls. Box and whiskers representation of log_2_ MFI values of antibodies against different *M*.*tb*. antigens. In each box plot, the central line inside the box represents the median. The box depicts the interval between 25% and 75% percentiles. Whiskers indicate the range of data spread while small circles show outliers. A. AFB^-^/Culture^-^ TB patient data (n = 23) are represented by red boxes, and blue boxes represent data for healthy individuals (n = 79), for each antigen. B. AFB^-^/Culture^-^ TB patient data (n = 23) are represented by red boxes, and green boxes represent data for COPD patients (n = 55), for each antigen.
(PDF)
