# Supplementary material for: Field evaluation of a blood based test for active tuberculosis in endemic settings
Source: PLoS One. 2017 Apr 5;12(4):e0173359. doi: 10.1371/journal.pone.0173359 (PMC5381859; doi:10.1371/journal.pone.0173359)
Supplement: S1 Table — (DOCX) [file pone.0173359.s001.docx]

**S1 Table**

**Summary of the sputum AFB microscopy and culture results in TB patients.**

| **Category** | **N** | **L J Culture^+^** | | **MGIT^+^**  **(AIMC only)**  **(%)** | **LJ^+^ and/or MGIT^+^**  **(%)** | **Culture^-^**  **At Both Labs (%)** |
| --- | --- | --- | --- | --- | --- | --- |
|  |  | **GDCH**  **(%)** | **AIMC**  **(%)** |  |  |  |
| **AFB^+^** | 100 | 98  (98) | 98  (98) | 98  (98) | 98  (98) | 2  (2) |
| **AFB^-^** | 124 | 80  (64) | 79  (63) | 79  (63) | 101  (81) | 23  (19) |
| **Total** | 224 | 178  (79) | 177  (79) | 177 (79) | 199  (89) | 25  (11) |
