# Supplementary material for: Field evaluation of a blood based test for active tuberculosis in endemic settings
Source: PLoS One. 2017 Apr 5;12(4):e0173359. doi: 10.1371/journal.pone.0173359 (PMC5381859; doi:10.1371/journal.pone.0173359)
Supplement: S2 Table — (DOCX) [file pone.0173359.s002.docx]

**S2 Table**

**Fold changes of antibodies in TB patients compared to controls by multivariate analysis.**

| **Antigen** | **Compared to Healthy** | | | | **Compared to COPD** | | | |
| --- | --- | --- | --- | --- | --- | --- | --- | --- |
|  | **AFB^+&-^**  **Cul^+^** | **AFB^+^**  **Cul^+^** | **AFB^-^ Cul^+^** | **AFB^-^ Cul^-^** | **AFB^+&-^**  **Cul^+^** | **AFB^+^**  **Cul^+^** | **AFB^-^ Cul^+^** | **AFB^-^ Cul^-^** |
| **A1** | 11.30^*^ | 15.56^*^ | 8.75^*^ | 4.32^*^ | 10.64^*^ | 14.62^*^ | 8.28^*^ | 4.06^*^ |
| **A2** | 12.02^*^ | 16.56^*^ | 8.75^*^ | 7.01^*^ | 9.28^*^ | 12.82^*^ | 6.73^*^ | 5.43^*^ |
| **A3** | 4.26^*^ | 4.35^*^ | 4.56^*^ | 2.58^**^ | 4.54^*^ | 4.63^*^ | 4.86^*^ | 2.73^**^ |
| **A4** | 7.45^*^ | 10.41^*^ | 5.28^*^ | 4.38^*^ | 7.75^*^ | 10.85^*^ | 5.46^*^ | 4.56^*^ |
| **A5** | 4.85^*^ | 5.39^*^ | 4.5^*^ | 4.08^*^ | 4.54^*^ | 5.06^*^ | 4.2^*^ | 3.81^*^ |
| **A6** | 3.51^*^ | 3.86^*^ | 3.46^*^ | 2.28^*^ | 2.17^**^ | 2.39^*^ | 2.14^*^ | 1.4 |
| **A7** | 1.95^*^ | 2.07^*^ | 1.93^*^ | 1.54 | 2.14^*^ | 2.28^*^ | 2.11^*^ | 1.68^**^ |
| **A8** | 8.00^*^ | 11.63^*^ | 5.43^*^ | 3.81^*^ | 8.16^*^ | 11.88^*^ | 5.54^*^ | 3.86^*^ |
| **A9** | 5.45^*^ | 6.63^*^ | 4.66^*^ | 3.86^*^ | 4.84^*^ | 5.9^*^ | 4.14^*^ | 3.43^*^ |
| **A10** | 4.33^*^ | 4.99^*^ | 3.94^*^ | 3.23^*^ | 4.01^*^ | 4.63^*^ | 3.63^*^ | 2.99^*^ |
| **A11** | 3.14^*^ | 3.34^*^ | 2.99^*^ | 2.97^*^ | 2.64^*^ | 2.81^*^ | 2.51^*^ | 2.5^*^ |
| **A12** | 2.54^*^ | 2.27^*^ | 2.95^*^ | 1.85 | 1.79 | 1.6 | 2.08^*^ | 1.31 |
| **A13** | 1.84^*^ | 1.66^*^ | 2.1^*^ | 1.49^**^ | 1.99^*^ | 1.79^*^ | 2.27^*^ | 1.61^**^ |
| **A14** | 3.31^*^ | 3.46^*^ | 3.29^*^ | 2.73^*^ | 2.98^*^ | 3.12^*^ | 2.97^*^ | 2.46^*^ |
| **A15** | 2.09^**^ | 2.19^*^ | 2.16^*^ | 1.34 | 2.09^**^ | 2.19^*^ | 2.16^*^ | 1.34 |
| **A16** | 2.96^*^ | 3.27^*^ | 2.85^*^ | 2.08^*^ | 2.78^*^ | 3.07^*^ | 2.68^*^ | 1.95^**^ |
| **A17** | 2.78^**^ | 3.1^*^ | 2.55^*^ | 2.39 | 0.79 | 0.88 | 0.72 | 0.68 |
| **A18** | 2.63^*^ | 2.71^*^ | 2.69^*^ | 2.04^*^ | 2.05^*^ | 2.11^*^ | 2.1^*^ | 1.59 |
| **A19** | 2.18^*^ | 2.1^*^ | 2.19^*^ | 2.43^*^ | 1.91^*^ | 1.85^*^ | 1.92^*^ | 2.14^*^ |
| **A20** | 2.84^*^ | 2.91^*^ | 2.77^*^ | 2.89^*^ | 1.90^*^ | 1.95^*^ | 1.85^*^ | 1.93^*^ |
| **A21** | 2.50^*^ | 2.58^*^ | 2.55^*^ | 1.95^*^ | 1.54^*^ | 1.59^*^ | 1.57^*^ | 1.2 |
| **A22** | 1.95^*^ | 1.99^*^ | 2.04^*^ | 1.38 | 2.45^*^ | 2.5^*^ | 2.57^*^ | 1.73^**^ |
| **A23** | 1.89^*^ | 1.74^*^ | 2.07^*^ | 1.71^*^ | 1.53^**^ | 1.41^**^ | 1.68^*^ | 1.38^*^ |
| **A24** | 3.92^*^ | 4.66^*^ | 3.32^*^ | 3.36^*^ | 2.95^*^ | 3.51^*^ | 2.5^*^ | 2.53^*^ |
| **A25** | 3.26^*^ | 3.27^*^ | 3.34^*^ | 2.87^*^ | 2.21^*^ | 2.22^*^ | 2.27^*^ | 1.95^**^ |
| **A26** | 1.58 | 1.55^*^ | 1.73^*^ | 1.02 | 1.72 | 1.69^*^ | 1.89^*^ | 1.11 |
| **A27** | 3.67^*^ | 2.85^*^ | 4.26^*^ | 4.56^*^ | 2.61^*^ | 2.03^*^ | 3.03^*^ | 3.25^*^ |
| **A28** | 5.39^*^ | 6.82^*^ | 4.5^*^ | 3.18^*^ | 4.33^*^ | 5.46^*^ | 3.63^*^ | 2.55^*^ |
| **A29** | 2.31^**^ | 2.19^*^ | 2.57^*^ | 1.65 | 1.31 | 1.25 | 1.46 | 0.94 |
| **A30** | 3.31^*^ | 4.06^*^ | 2.6^*^ | 3.25^*^ | 2.92^*^ | 3.58^*^ | 2.3^*^ | 2.85^*^ |
| **A31** | 2.71^*^ | 3.03^*^ | 2.58^*^ | 1.87^*^ | 1.20 | 1.34 | 1.15 | 0.82 |

**Significance:** ^*^p-Values below 0.01; ^**^p-Values below 0.05
