# Supplementary material for: Field evaluation of a blood based test for active tuberculosis in endemic settings
Source: PLoS One. 2017 Apr 5;12(4):e0173359. doi: 10.1371/journal.pone.0173359 (PMC5381859; doi:10.1371/journal.pone.0173359)
Supplement: S3 Table — (DOCX) [file pone.0173359.s003.docx]

**S3 Table**

**Performance of six classification algorithms compared to optimized Decision Tree.**

| **Algorithm** | **SN**  **(%)** | **PPV**  **(%)** | **NPV**  **(%)** | **SP (COPD)**  **(%)** | **SP (Healthy)**  **(%)** | **TE**  **(%)** | **MCC**  **(%)** |
| --- | --- | --- | --- | --- | --- | --- | --- |
| **Support Vector Machine** | 78.81 | 93.37 | 72.33 | 94.55 | 87.34 | 83.14 | 67.26 |
| **Naïve Bayes** | 72.83 | 94.08 | 67.56 | 92.73 | 91.14 | 80.32 | 63.44 |
| **k-Nearest Neighbor** | 74.32 | 91.44 | 67.41 | 87.27 | 84.81 | 79.49 | 60.54 |
| **Logistic Regression** | 80.58 | 91.45 | 73.22 | 87.27 | 84.81 | 83.15 | 66.33 |
| **Decision Tree** | 88.33 | 87.55 | 80.07 | 80.00 | 74.68 | 84.82 | 67.34 |
| **Random Forest** | 87.86 | 88.04 | 79.64 | 80.00 | 75.95 | 84.83 | 67.45 |
| **Optimized Decision Tree** | 90.09 | 95.69 | 85.03 | 96.36 | 91.14 | 91.29 | 82.04 |

SN=Sensitivity, PPV=Positive Predictive value, NPV= Negative Predictive Value, SP=Specificity, TE =Test Efficiency, and MCC= Mathew Correlation Coefficient
