# Supplementary material for: Field evaluation of a blood based test for active tuberculosis in endemic settings
Source: PLoS One. 2017 Apr 5;12(4):e0173359. doi: 10.1371/journal.pone.0173359 (PMC5381859; doi:10.1371/journal.pone.0173359)
Supplement: S4 Table — (DOCX) [file pone.0173359.s004.docx]

**S4 Table**

**Comparison of sensitivity and specificity of the Multiplex TB Serodiagnostic Panel containing 11 antigens to established sputum-based tests.**

|  | Multiplex TB Panel (RU-1)  (11 antigens) | Microscopy (AFB) | Culture | Cepheid Xpert MTB/RIF ([17](#_ENREF_17)) |
| --- | --- | --- | --- | --- |
| Sensitivity (AFB^+^) | 95% | 100% | 98-100% | 98% |
| Sensitivity (AFB^-^/culture^+^) | 88% | 0% | 100% | 72% |
| Sensitivity (AFB ^+^ and AFB^-^, culture^+^) | 91% | 30-70% | 100% | 92% |
| Specificity (COPD) | 96% | 99% | 98% | 99% |
| Time to diagnosis | 2 hours | 2-3 days | 2-8 weeks | 2 hours |
| Patient sample source | Blood | Sputum | Sputum | Sputum |
| Throughput/day (patients) | 360^1^ | 20 slides/tech^2^ | -- | 20 |

^1^Throughput based on a single instrument in an 8-hour day

^2^WHO recommended maximum slides to be viewed by a single technician per day
