# Supplementary material for: Field evaluation of a blood based test for active tuberculosis in endemic settings
Source: PLoS One. 2017 Apr 5;12(4):e0173359. doi: 10.1371/journal.pone.0173359 (PMC5381859; doi:10.1371/journal.pone.0173359)

S2 Fig

Anti-*M. tb.* antibody levels in TB patients compared to controls.

A. **AFB$^-$/Culture$^+$ *Vs.* Healthy**

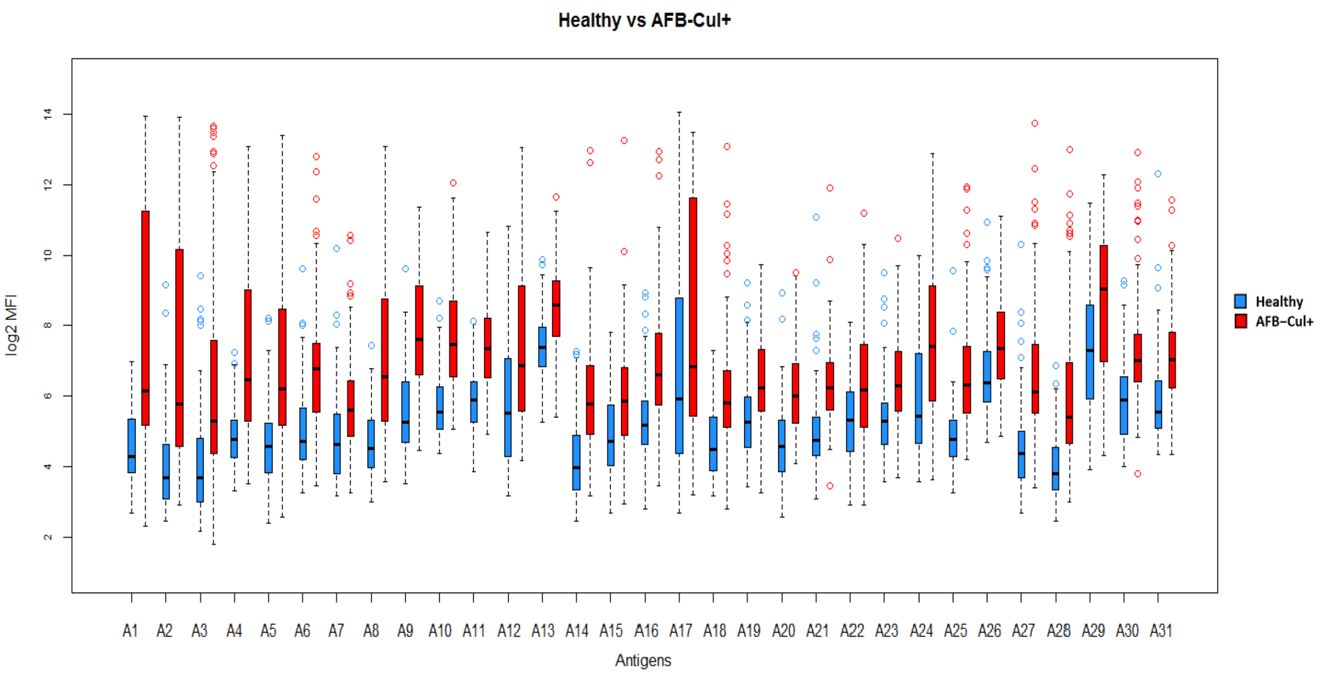

B. **AFB$^-$/Culture$^+$ *Vs.* COPD**

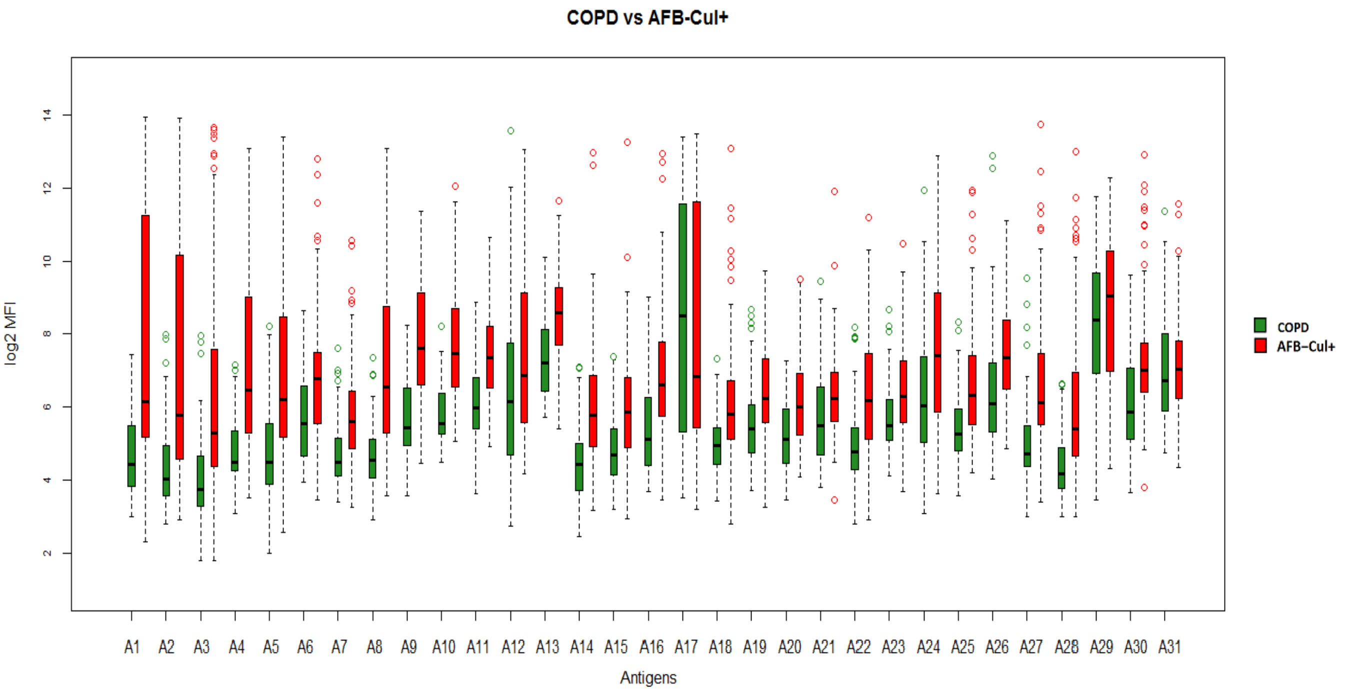

Supplement: S2 Fig — Box and whiskers representation of log2 MFI values of antibodies against different M.tb. antigens. In each box plot, the central line inside the box represents the median. The box depicts the interval between 25% and 75% percentiles. Whiskers indicate the range of data spread while small circles show outliers. A. AFB-/Culture+ TB patient data (n = 101) are represented by red boxes, and blue boxes represent data for healthy individuals (n = 79), for each antigen. B. AFB-/Culture+ TB patient data (n = 101) are represented by red boxes, and green boxes represent data for COPD patients (n = 55), for each antigen. (PDF) [file pone.0173359.s007.pdf]
